# The Fat Body Transcriptomes of the Yellow Fever Mosquito *Aedes aegypti*, Pre- and Post- Blood Meal

**DOI:** 10.1371/journal.pone.0022573

**Published:** 2011-07-27

**Authors:** David P. Price, Vijayaraj Nagarajan, Alexander Churbanov, Peter Houde, Brook Milligan, Lisa L. Drake, John E. Gustafson, Immo A. Hansen

**Affiliations:** 1 Department of Biology, New Mexico State University, Las Cruces, New Mexico, United States of America; 2 The Institute of Applied Biosciences, New Mexico State University, Las Cruces, New Mexico, United States of America; 3 The Molecular Biology Program, New Mexico State University, Las Cruces, New Mexico, United States of America; 4 The Roadrunner Sequencing Lab, New Mexico State University, Las Cruces, New Mexico, United States of America; 5 Bioinformatics and Computational Biosciences Branch (BCBB), OCICB/OSMO/OD/NIAID/NIH, Bethesda, Maryland, United States of America; Universidade Federal do Rio de Janeiro, Brazil

## Abstract

**Background:**

The fat body is the main organ of intermediary metabolism in insects and the principal source of hemolymph proteins. As part of our ongoing efforts to understand mosquito fat body physiology and to identify novel targets for insect control, we have conducted a transcriptome analysis of the fat body of *Aedes aegypti* before and in response to blood feeding.

**Results:**

We created two fat body non-normalized EST libraries, one from mosquito fat bodies non-blood fed (NBF) and another from mosquitoes 24 hrs post-blood meal (PBM). 454 pyrosequencing of the non-normalized libraries resulted in 204,578 useable reads from the NBF sample and 323,474 useable reads from the PBM sample. Alignment of reads to the existing reference *Ae. aegypti* transcript libraries for analysis of differential expression between NBF and PBM samples revealed 116,912 and 115,051 matches, respectively. *De novo* assembly of the reads from the NBF sample resulted in 15,456 contigs, and assembly of the reads from the PBM sample resulted in 15,010 contigs. Collectively, 123 novel transcripts were identified within these contigs. Prominently expressed transcripts in the NBF fat body library were represented by transcripts encoding ribosomal proteins. Thirty-five point four percent of all reads in the PBM library were represented by transcripts that encode yolk proteins. The most highly expressed were transcripts encoding members of the cathepsin b, vitellogenin, vitellogenic carboxypeptidase, and vitelline membrane protein families.

**Conclusion:**

The two fat body transcriptomes were considerably different from each other in terms of transcript expression in terms of abundances of transcripts and genes expressed. They reflect the physiological shift of the pre-feeding fat body from a resting state to vitellogenic gene expression after feeding.

## Introduction

The yellow fever mosquito, *Aedes aegypti*, is the primary vector for dengue fever, several encephalitis viruses, yellow fever, and several types of filariasis [1]. Due to its ability to transmit diseases and its widespread range, this disease vector continually affects the health of millions of people around the globe [2].

The fat body is the principal organ of intermediary metabolism, functioning as a storage unit for lipids, carbohydrates, and proteins in both mosquitoes and insects in general. The principal cell type of the fat body is a large polyploid cell referred to as the trophocyte, which is capable of synthesizing large amounts of protein and contains many ribosomes and oil droplets [3]. The fat body also acts as the main source of hemolymph proteins in all developmental stages of holo-, hemi-, and a-metabolic insects [4]. Metabolic capabilities that differ among regions within the fat body have been reported for several lepidopteran[5], [6] and one dipteran species [7], but these regional metabolic differences are generally not well understood.

During the mosquito aquatic larval stage, the fat body accumulates and stores nutrients for use in the adult stage. Hexameric storage proteins of the arylphorin family are the major amino acid stores in mosquito larvae and are synthesized by the fat body and secreted into the hemolymph. These hexamerins are then reabsorbed later by the larval fat body via receptor-mediated endocytosis, stored in protein granula, and afterward hydrolyzed to deliver energy and building blocks required to drive metamorphosis into the adult insect [8], [9].

In the adult stage the fat body continues to play a prominent role in energy metabolism by providing precursors for flight and yolk protein synthesis which is required by females for reproduction. It also assists in metabolizing potentially fatal amounts of excess ammonia after the female takes a blood meal [10]. In addition, the fat body plays an important role in several immune pathways and antimicrobial peptide production which control and prevent infection by microbial and protozoan pathogens [11]. It has been demonstrated several times that altering mosquito fat body immune function via transgenic interventions drastically alters vectorial capacity by resulting in overly pathogen-susceptible or -resistant mosquitoes [12].

Before a female mosquito takes a blood meal, her fat body is in a state-of-arrest with regards to the expression of genes involved in reproduction. When a blood meal is taken, a sequence of signals originating in other tissues affects the fat body, priming it for gene expression alterations. These signals include raised amino acid levels in the hemolymph, peptide hormones from the gut and the central nervous system [13], and ecdysteroids from the ovaries [14], [15], [16]. Additional signaling molecules present in the blood meal itself (e.g., vertebrate insulin) have been shown to alter mosquito fat body metabolism [17]. Collectively, these signals can activate the insulin and target of rapamycin signaling pathway within the fat body, which leads to the production of yolk protein precursors [14], [16], [18], [19], [20]. During vitellogenesis, yolk proteins and lipids are secreted by the fat body trophocytes and transported to the ovaries which deposit them into developing eggs [21].

The adult fat body is a key organ in mosquito reproduction and immunity; therefore, it is important to understand fat body physiology on a molecular level. We now report on transcriptional alterations occurring in *Ae. aegypti* fat body tissue following a blood meal. This was accomplished by comparing and contrasting read and contig libraries, obtained by 454 pyrosequencing, derived from fat body tissue of female non-blood fed (NBF) *Ae. aegypti* and 24 h post-blood meal (PBM) fat body tissue. This study describes a window of transcriptional alterations appearing in the *Ae. aegypti* fat body during the digestion of a blood meal and subsequent accumulation and utilization of nutrients. In particular we used an RNA-seq approach to observe transcript expression transitions from a resting fat body prepared for translational activity to a fat body producing transcripts involved in *Ae. aegypti* vitellogenesis.

## Results and Discussion

Twenty four hours after a full blood meal, vitellogenesis, the production, secretion, and reuptake of yolk proteins, is at a peak in *Ae. aegypti* [22]. Due to the relationship of this process with fat body metabolism, in this study we choose to compare the fat body tissue transcriptomes of NBF mosquitoes with fat body tissue of mosquitoes 24 hrs after feeding.

For practical reasons we isolated abdominal body walls with attached abdominal fat body tissue and processed them for library construction. This is a standard preparation for studying mosquito fat body physiology [14], [18], [23]. It must be noted that this fat body preparation contains other cells and tissues besides fat body trophocytes for example epidermis, tracheas, muscle, and ventral nerve cord. However, the fat body is the dominant tissue in this preparation and there is little doubt that the transcripts we discuss below are expressed in the fat body.

### Alterations in total RNA quantities in the fat body tissue following feeding

It has been shown previously that there is a large increase in RNA synthesis and accumulation in the PBM fat body compared to the NBF fat body [24]. As expected, comparing total RNA isolated from fat bodies (4 samples per treatment consisting of 5 fat bodies each) in NBF and PBM mosquitoes revealed an average of 2.1 µg of RNA per NBF fat body and 4.7 µg per PBM fat body (p<0.005, students t-test). These results indicate that the total amount of RNA in the fat body doubles during the 24 hrs following a blood meal. Based on previous findings [24], we speculate that these differences in RNA accumulation may reflect the increased transcription of ribosomal structural RNAs in the PBM fat body.

### 454 pyrosequencing and contig assembly

The NBF and PBM fat body libraries were derived by 454 sequencing: a total of 40 and 55 megabases were read, consisting of 204,578 and 323,474 individual reads averaging 194 and 171 bases in length, respectively. Sequence quality reported by the sequencer for our NBF and PBM sequences using the phred quality scale [25], [26] revealed that the average quality was 32.9 and 33.1 (equivalent to >99.9% base call accuracy), respectively. Therefore, over half a million high quality *Ae. aegypti* fat body reads were generated in this study (for size distribution of library reads see Figure S1 A & B). All sequences generated were submitted to the sequence read archive [27] and accepted under accession number SRA024707.1.

The individual reads were *de novo* assembled using Mira assembly software [28]. The assembly process produced 15,456 and 15,010 contigs from the NBF and PBM sample reads, respectively (Table 1). These contigs were used in our analyses. Of the total NBF and PBM contigs, 11,588 and 11,129 respectively, qualified as “true contigs”, which were assembled out of at least two reads each. The average GC content of the NBF contigs is 47.5% and the PBM contig populations is 46.9% (See Figures S2 A and B). These numbers are comparable with other insect and eukaryote sequencing projects, which have reported GC content between 38.7% and 56.5% [29], [30], [31], [32].

**Table 1:** Fat body Contig Statistics.

Sample	Average read length	Average reads per contig	Average contig length (bases)	Maximum contig length (bases)	Minimum contig length
NBF	194.4	3.65	382.85	1439	44
PBM	170.8	3.79	379.74	2288	40

### Gene Ontology (GO) analysis

The Blast2GO [33], [34] program, which uses a pipeline of BlastX followed by GO term assignment and annotation, was utilized to analyze gene ontology (GO) and categorize fat body expressed genes in the NBF and PBM contig samples. A sanity check of the contigs was performed and, as expected, the overwhelming majority of contigs with BlastX results for both NBF and PBM libraries exhibited greatest similarity to protein sequences from *Ae. aegypti* or other mosquito species (Figures S3 A and B). Statistics on NBF and PBM library contig numbers passing each stage of Blast2GO are shown in Table 2.

**Table 2:** Results of contig processing with Blast2GO.

	NBF (#contigs)	PBM (#contigs)
Annotated, GO terms assigned, Blastx results	5925	5091
GO terms assigned, Blastx results	325	412
Blastx results	748	797
No Blastx results	8458	8710
Totals	15456	15010
True Contigs	11588	11129

**Figure 1:**
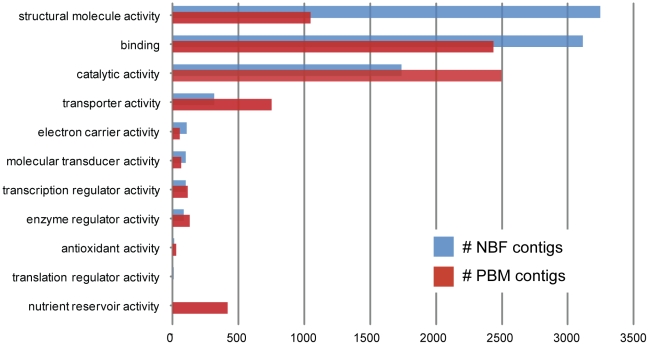
Gene Ontology of the *Aedes aegypti* fat body. Pre-and Post-Bloodmeal Level 2 GO functions for *de novo* assembled contigs. The x-axis represents the total number of contigs with the given level 2 GO term.

Comparison of the level two GO functions of the two libraries (see Figure 1) shows that contigs with transporter, catalytic and nutrient reservoir activities are much more prevalent in the PBM than in NBF library. In contrast, contigs with binding and structural molecule activity functions, including all ribosomal proteins, are heavily reduced in the PBM compared to NBF library. Based on these results, we suggest that fat body gene expression shifts from the production of transcripts associated with the protein translation machinery to the expression of transcripts involved with nutrient accumulation and utilization during vitellogenesis.

### Novel transcript discovery

The fat body contig libraries generated in this study include a collection of potential transcripts not previously identified in the *Ae. aegypti* genome sequence in Vectorbase [35]. We identified a total of 123 of these, which are listed in Table S1. Examples of some of these potential transcripts are shown in Table 3. The majority of these contigs mapped to the reference genome where there was no overlap with existing predicted genes. However, there were exceptions. For instance, contig 6072 (Table 3) which, based on blast results, encodes a putative sugar transporter, overlaps with the gene encoding 4-hydroxyphenyl-pyruvate dioxygenase (AAEL014600). While presently unclear, overlapping contigs might be explained by a variety of mechanisms (e.g. alternative splicing, pre-splice RNA, overlapping genes).

**Table 3:** Twenty potential new genes identified using contigs.

Contig	Len	Reads	Ref Contig	Mlen	Mis	Gaps	Blastx results	Accession	e-val	domains	EST
48	382	31	1.971	291	0	0	hypothetical protein *Tetrahymena thermophila*	XP_977125.1	3.2	LC	1E-163
							hypothetical protein AaeL_AAEL000019	XP_001647901.1	7.1		
114	511	12	1.329	425	3	0	sodium/potassium/calcium exchanger 5 precursor, putative (*Pediculus humanus corporis*)	XP_002429936.1	7.1	LC, TM	0
							similar to potassium-dependent sodium-calcium exchanger (*Acyrthosiphon pisum*)	XP_003243863.1	9.3		
808	654	8	1.778	584	0	1	hypothetical protein CpipJ_CPIJ018486(Culex quinquefasciatus)	XP_001868660.1	2.E-06	SP, LC, TM	0
							vitelline membrane protein 32E (*Drosophila erecta*)	ABO71727.1	0.034		
836	576	7	1.177	495	0	0	chaperonin (*Culex quinquefasciatus*) >gb|EDS29066.1|	XP_001869348.1	0.29	BT, CC, LC	3E-07
1514	528	3	1.495	439	4	1	ribosomal protein S27 (*Aedes albopictus*)	AAV90719.1	8E-32	RS27e, BT	0
1602	463	7	1.363	352	0	2	no sig				1E-18
1651	569	4	1.55	521	1	6	conserved hypothetical protein (*Culex quinquefasciatus*)	XP_001848093.1	2E-38	BT	3.E-04
							AGAP010003-PA (*Anopheles gambiae str. PEST*)	XP_319147.4	2E-29		
2468	529	3	1.389	474	3	2	heat shock protein 70 B2 (*Culex quinquefasciatus*)	XP_001864723.1	2E-32	TM, LC, EGF(BT)	0
							heat shock protein 70 (*Chironomus riparius*)	ADL27420.1	9E-32		
2644	665	3	1.563	622	1	3	Os02g0274400 (*Oryza sativa Japonica* Group)	NP_001172895.1	5.4	LC, BT	1.E-04
3974	280	4	1.133	232	2	1	NADH dehydrogenase subunit 6 (*Aedes aegypti*)	YP_001649172.1	1.E-06	TM, BT	3E-09
4010	350	3	1.102	281	1	0	Lian-Aa1 retrotransposon protein (*Aedes aegypti*)	AAB65093.1	5E-14	BT	1E-156
4015	332	3	1.363	310	0	1	cytochrome c oxidase subunit II (*Aedes aegypti*)	YP_001649164.1	5E-43	COX2	1E-171
5669	448	2	1.349	363	2	2	response regulator receiver domain-containing protein(*Methanospirillum hungatei*)	YP_502925.1	0.49	LC, BT	2.E-05
							hypothetical protein Phum_PHUM231450(*Pediculus humanus corporis*)	XP_002426007.1	0.64		
6072	227	2	1.119	187	0	1	sugar transporter (*Culex quinquefasciatus*)	XP_001846219.1	9.3	BT	5E-98
6085	885	3	1.371	701	1	2	Senescence-associated protein (*Brugia malayi*)	XP_001900327.1	1E-34	BT	0
							Uncharacterized protein ART2 (*Camponotus floridanus*)	EFN65036.1	6E-33		
6816	420	2	1.12	329	1	2	hypothetical protein CpipJ_CPIJ015859(*Culex quinquefasciatus*)	XP_001865960.1	1E-11	SP, LC, TM, BT	1E-179
8377	484	2	1.68	392	1	1	heat shock 70 Ba (*Aedes aegypti*)	ACJ64195.1	6E-12	LC, BT	0
9356	769	2	1.495	626	1	5	40S ribosomal protein S27 (*Culex quinquefasciatus*)	XP_001847201.1	2E-44	LC, SP, TM, BT	0
11935	227	1	1.15	196	1	0	cytosolic large ribosomal subunit L27A(*Ochlerotatus taeniorhynchus*)	ACJ74464.1	6E-12	L15, BT	1E-106
14965	497	2	1.115	398	3	3	putative salivary odorant binding protein 1(*Culex quinquefasciatus*)	AAR18408.1	1E-07	BT	0

Contig name, length of contig, number of reads making up the contig, *Ae. aegypti* reference genome supercontig our contig matched against, length of the match, number of base mismatches, number of base gaps, results from NCBI Blastx search, eval of Blastx result, pfam/SMART domains identified with eval greater than 1e-5, e-val of best EST hit at vectorbase. LC - low complexity, TM – transmembrane, BT - below threshold, RS27e - ribosomal S27e, SP - signal peptide, CC - coiled coil, IR - internal repeat, EGF - epidermal growth factor, COX2- Cytochrome C oxidase subunit II, L15 – Ribosomal protein L15.

### Transcript expression in the fat body

Alignment of our NBF and PBM transcriptome sequencing reads to the Vectorbase *Ae. aegypti* reference transcripts produced a total of 116,912 and 115,051 alignments, respectively. These alignments covered 6,019 (NBF) and 7,625 (PBM) reference transcripts. The top 4 transcripts aligned with on a percentage basis from each sample are shown in Table S2 and all alignments are shown in Table S3. Table S4 shows all transcripts found to exhibit a statistically significant change in expression and the fold difference in expression between the unfed and bloodfed state in our libraries. The union of these two sets produced a set of 9,984 transcripts, representing all transcripts, including multiple isoforms, found to be expressed in either the NBF or PBM fat body tissue.

Of these 9,984 distinct transcripts, 4,974 exhibited at least a 2-fold change in expression level. This is substantially lower than the number of differentially expressed genes previously reported (8,288 based on several repeats, but no statistical filter applied to changes in transcript levels) in NBF vs 24 hr PBM whole mosquitoes examined via microarray analysis [36]. A major contributor to the differences between these two studies may be the difference in tissues: previously, the whole mosquito was examined whereas only fat body/abdomen tissue was examined in this study. It is highly likely that blood feeding affects the entire organism at a transcriptional level, in different ways than the fat body . Statistical analysis of our results [37], [38] revealed that 236 (2.4% of all) transcripts our reads aligned with had significant differential expression between samples.

When all isoforms of each transcript are counted as one, we identified 2,354 NBF and 2,746 PBM transcripts from the total of 6,019 and 7,625 distinct transcript isoforms. These numbers represent estimates of the number of genes expressed in the fat body under each physiological condition. They are comparable to previous *Drosophila melanogaster* fat body gene expression estimates (2261+ genes) [39], [40].

### Transcripts highly expressed in NBF fat body tissue

The condition of the mosquito fat body before the female takes a blood meal has been described as a “previtellogenic state-of-arrest” during which yolk proteins are not expressed [41]. Fifteen of our highly expressed transcripts found in our state-of-arrest NBF fat body library (Table 4) encode ribosomal proteins (22% of read alignments with reference transcriptome) and one transcript encodes the eukaryotic translation elongation factor. The last four of our highly expressed NBF transcripts potentially encode serine hydroxymethyltransferase, NADH dehydrogenase subunit 2, and two proteins of unknown function. All transcripts shown in Table 4, with the exception of NADH dehydrogenase subunit 2, were found to have statistically significant differential expression.

**Table 4:** Top 20 most highly expressed transcripts in fat body of non-blood fed mosquitoes.

Ensembl TranscriptID	Name	NBF	PBM	Change	R-val	% Pre	% Post
AAEL009341	60S ribosomal protein L34	2189	938	0.44	107.5	3.6	1.5
AAEL013221	60S ribosomal protein L10a	2065	673	0.33	156.3	3.4	1.1
AAEL001849	60S ribosomal protein L34	2036	859	0.43	102.9	3.4	1.4
AAEL004149	unknown membrane protein	868	398	0.47	37.2	1.4	0.6
AAEL003877	ubiquitin/ribosomal protein L40	796	444	0.57	20.8	1.3	0.7
AAEL011471	60S ribosomal protein L17	737	220	0.3	62.2	1.2	0.4
AAEL002510	serine hydroxymethyltransferase	739	194	0.27	71.8	1.2	0.3
AAEL000032	40S ribosomal protein S6	699	232	0.34	51.6	1.2	0.4
AAEL013158 AAEL005901	40S ribosomal protein S3a	696	331	0.48	27.5	1.1	0.5
AAEL017516	60S ribosomal protein L23a	693	276	0.4	38.8	1.1	0.4
AAEL000823	60S ribosomal protein L35A	566	235	0.42	29.5	0.9	0.4
AAEL006698	60S ribosomal protein L31	560	238	0.43	27.9	0.9	0.4
AAEL003530 AAEL005027	acidic ribosomal protein P1	543	153	0.29	49.0	0.9	0.3
AAEL017413	NADH dehydrogenase subunit 2	511	560	1.11	0.7	0.8	0.9
AAEL004175	40S ribosomal protein S17	504	192	0.39	30.4	0.8	0.3
AAEL008103	40S ribosomal protein S8	498	215	0.44	24.1	0.8	0.4
AAEL004500	eukaryotic translation elongation factor 2	478	216	0.46	21.1	0.8	0.4
AAEL004851	Unknown protein	474	187	0.4	27.0	0.8	0.3
AAEL012686	40S ribosomal protein S12	470	172	0.37	30.2	0.8	0.3
AAEL008188	60S ribosomal protein L6	467	181	0.39	27.4	0.8	0.3

Reference transcripts by number of reads aligned in the pre-blood meal sample. NBF, number of reads aligning to the shown transcript ID in pre-blood meal sample. PBM, number of reads aligning in the post-blood meal sample. Change is the number of PBM reads divided by the number of reads in the NBF sample, with normalization. Normalization was accomplished by multiplying the number of reads PBM by the ratio of the total number of NBF reads divided by the total number of PBM reads. R-val is the computed R-value, R>9 is significant. %Pre is the percentage of unique alignments in the NBF sample the NBF alignments for this transcript represents. %Post is the same for the PBM sample.

Cytosolic serine hydroxymethyltransferase is found in eukaryotes and prokaryotes. It is a central enzyme of the one-carbon metabolic pathway that catalyzes the production of the major one-carbon donors for the biosynthesis of thymidylate, purines, methionine and choline [42]. NADH dehydrogenase, also called Complex I, is a protein localized in the inner mitochondrial membrane and the first protein of the oxidative phosphorylation process in mitochondria. The eukaryotic translation elongation factor 2 is part of the ribosomal protein synthesis machinery.

The strong presence of ribosomal protein transcripts in the NBF library was expected since these messages have been shown to accumulate in the fat body during the first days after eclosion of adult *Ae. aegypti* [43]. Translation of ribosomal proteins, transcription of rRNAs, and ribosome assembly start directly after a blood meal resulting in ribosome accumulation in the fat body. After completion of vitellogenesis most of these ribosomes degrade and the number of ribosomes returns to pre-blood meal levels.

Our data support the finding of Niu and Fallon [43] that ribosomal protein (rp) L34 is down-regulated after a blood meal. In fact, the number of all transcripts encoding ribosomal proteins are reduced in the PBM library (see Table 5). Interestingly, two transcripts encode rpL34 homologues (AAEL009341, AAEL001849). These two proteins have an identity of 68% when aligned and make up for 7% of all aligned transcripts in the NBF library.

**Table 5:** Top 20 most highly expressed cDNAs PBM, reference transcripts by number of reads aligned in the PBM sample.

Ensembl TranscriptID	Name, references	NBF	PBM	Change	R-val	% Pre	% Post
AAEL007585AAEL012216AAEL015312	VCB-A [15], [56], [78]	7	4120	598	1234.3	0.01	6.6
AAEL006138	vitellogenin-B [15]	14	3306	240	971.6	0.02	5.3
AAEL010434	Vitellogenin-A [15]	13	3194	250	939.8	0.02	5.1
AAEL007599	VCB-B [15], [56], [78]	1	2507	2548	760.0	0.002	4.0
AAEL006126	vitellogenin-C [15]	6	2141	363	635.9	0.01	3.5
AAEL007590	VCB-C [15], [56], [78]	3	1936	656	580.7	0.005	3.1
AAEL006563	VCP-A [50]	4	1721	437	513.0	0.007	2.8
AAEL009341	ribosomal protein L34	2189	938	0.44	107.5	3.6	1.5
AAEL001849	60S ribosomal protein L34	2036	859	0.43	102.9	3.4	1.4
AAEL006542	VCP-B [50]	3	806	273	237.8*	0.005	1.3
AAEL014561	vitelline membrane protein homolog [52]	0	699	n/a	212.9*	0	1.1
AAEL013221	ribosomal protein L10a	2065	673	0.33	156.3	3.4	1.1
AAEL017413	NADH dehydrogenase subunit 2	511	560	1.11	0.7	0.8	0.9
AAEL003877	Ubiquitin precursor	796	444	0.57	20.8	1.3	0.7
AAEL013027	vitelline membrane protein [52]	0	403	n/a	122.7*	0	0.7
AAEL004149	unknown membrane protein	868	398	0.47	37.2	1.4	0.6
AAEL009637	VCB-D [15], [56], [78]	73	383	5.33	51.3	0.1	0.6
AAEL006670	vitelline membrane protein	0	371	n/a	113.0*	0	0.6
AAEL003345	argininosuccinate lyase	263	365	1.41	4.0	0.4	0.6
AAEL013158	ribosomal protein S3a	696	331	0.48	27.5*	1.2	0.5

NBF, number of reads aligning to the shown transcript ID in NBF sample. PBM, number of reads aligning in the post-blood meal sample. Change is number of post-BM reads divided by number of reads in the NBF sample, with normalization. Normalization was accomplished by multiplying the number of reads PBM by the ratio of the total number of NBF reads divided by the total number of PBM reads. R-val is the computed R-value, R>9 is significant. %Pre is the percentage of unique alignments in the NBF sample the number of alignments in this sample represents. %Post is the same for the PBM sample.

* Also significant by DESeq results (p<0.05).

### Transcripts highly expressed in PBM fat body tissue

After a blood meal the fat body is activated and starts vitellogenic gene expression [21]. Several signals, including the rise of hemolymph amino acids (coming from the midgut), the steroid hormone ecdysone (secreted by the ovaries) and insulin-like peptides (from the central nervous system), have been identified as regulators of yolk protein expression [14], [15], [16]. At 24 h PBM the process of vitellogenesis peaks and the fat body produces large amounts of yolk proteins that are secreted into the hemolymph. The 20 most highly expressed transcripts found in the 24 h PBM library are presented in Table 5. All transcripts in Table 5, with the exception of NADH dehydrogenase subunit 2 were found to have differential expression.

#### Vitellogenins

Vitellogenin is the principal yolk protein precursor in almost all oviparous vertebrates and invertebrates [44], [45]. It is a nutrient-rich glycolipoprotein that gives rise to vitellin, the major yolk protein in eggs. Vitellogenins A, B and C (AAEL010434, AAEL006138, AAEL006126) were found to have differential expression between NBF and PBM samples.

#### Vitellogenic cathepsin

Cathepsins are cysteine thiol proteases, usually located in the lysosomes, which are found in a wide variety of organisms ranging from humans, rats and cattle to papaya (as papain [46]) and insects. These proteases have a wide variety of described functions: fetal myotube development, normal and tumor angiogenesis, digestion, and vitellogenesis [47], [48], [49], [50], [51]. In *Ae. aegypti*, vitellogenic cathepsin b (VCB) is secreted into the hemolymph as 44 kDa subunits. It is taken up by oocytes and processed during several stages of vitellogenesis. Finally, it becomes a 33 kDa form during embryogenesis. This is considered the active form and degrades vitellogenin [49].

**Figure 2:**
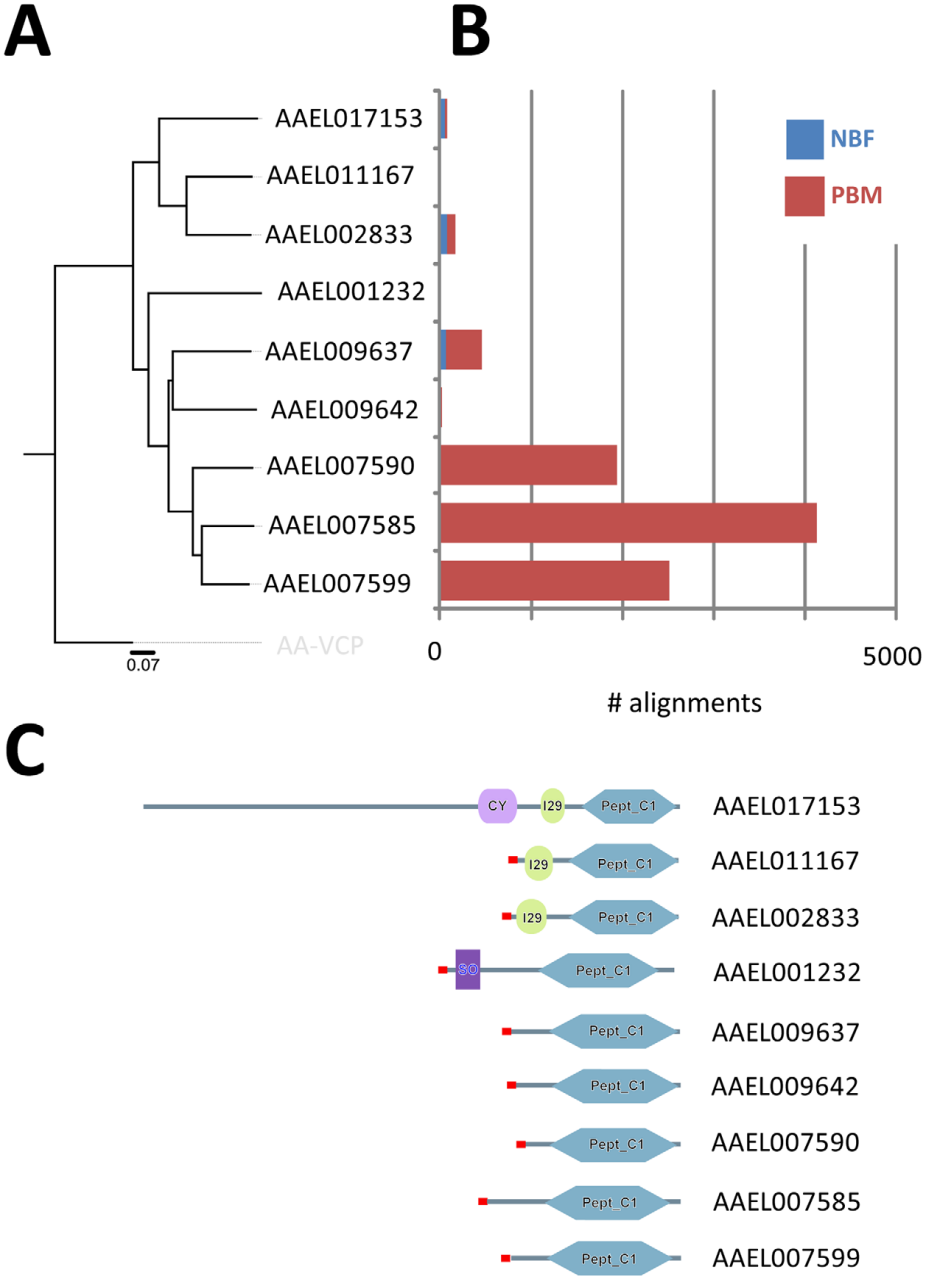
The VCB family of *Aedes aegypti*. **A**. Neighbor joining tree showing evolutionary relationships of *Ae. aegypti* VCBs. **B**. Number of VCB reads identified in the NBF and PBM libraries. All cathepsins identified in *Ae. aegypti* which had reads align using the methods used for sequence alignment and data analysis, are represented. **C**. Domain structure of *Ae. aegypti* VCB proteins. Cy - Cystatin-like domain, Pept_C1 - Papain family cysteine protease domain, I29 - Cathepsin propeptide inhibitor domain, SO - Somatomedin B -like domain. Signal peptides are labeled red.

We found three cathepsin transcripts (AAEL007590, AAEL007585, AAEL007599) to be heavily up-regulated in the PBM fat body and a fourth (AAEL009637) that appears to have a lesser degree of up-regulation (Figure 2A & B). The changes in expression levels for all four were found to be statistically significant. Our phylogenetic analysis shows AAEL007599 and AAEL007585 to be the most closely related while AAEL009637 diverges the most from this small group. Analysis using SMART software [52] shows that all four of these VCBs have an identical protein domain organization with a signal peptide followed by a single cysteine protease domain (Figure 2 C).

#### Vitellogenic carboxypeptidase (VCP)

We observed VCP to be one of the most up-regulated genes in the fat body after a blood meal. Serine carboxypeptidases are found in many different species, from invertebrates to humans, and in many cell types in those organisms. The described function for the serine carboxypeptidase family is the removal of one or more amino acids from the carboxy terminal end of an amino acid chain [53], [54]. In *Ae. aegypti*, vitellogenic carboxypeptidase (VCP) has been found to be transcribed and translated in the fat body and exported to the developing oocytes after a blood meal, with the peak of transcription at 24 hours post blood meal and very little transcription by 48 hours post blood meal [55]. Once in the oocytes, VCP surrounds the vitellin yolk in the same manner as cathepsin B [53], [56]. VCP is modified from a 53 kDa form to a 48 kDa form at the onset of embryogenesis and is rendered into small amino acid sequences by the time the embryo reaches the first instar. The function of VCP has not been described to our knowledge. Similar carboxypeptidases have been shown to have a role activating or modifying other enzymes and molecules, but it is not thought to act upon vitellogenin [57].

#### Vitelline membrane proteins (VMP)

VMPs are an important part of the vitellogenic process, forming the inner layer of the *Ae. aegypti* eggshells. We were surprised to find them highly expressed in the vitellogenic fat body, but we were able to confirm these results via RT-PCR repeatedly (data not shown). VMPs have been described as being exclusively secreted by the follicular epithelium [58], but there is some evidence that they are also produced in the fat body. We also found ESTs encoding VMPs in a collection of *Ae. aegypti* fat body specific ESTs within the Unigene and dbEST database [59], [60]. We found transcripts for AAEL006670, AAEL013027 and AAEL014561 to be highly upregulated post blood-meal.

### Fat body membrane transporters

**Figure 3:**
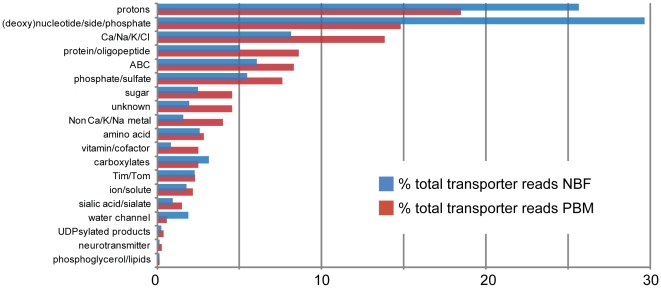
Transporters identified in the *Aedes aegypti* fat body. Number of reads in the NBF and PBM samples by transporter type. Transporters were identified as described in the text and the number of reads aligned were pulled from the aligned reads generated for our EST expression in the fat body overview.

Since the fat body is a key player in mosquito metabolism, efficient transport across its cell membrane is extremely important for homeostasis. The mosquito fat body must import nutrients derived from a blood meal for the rapid and energetically costly process of vitellogenesis. A wide variety of transporter families capable of transporting a plethora of substrates have been identified by our sequencing. We mapped reads to 54 out of 58 of the families represented by the list of transporters we obtained. Figure 3 shows the total number of transporter reads aligned by substrate transported.

The total number of transporter reads decreases PBM by approximately one third. This is approximately the number of reads taken up by yolk protein reads in the PBM library, which may reduce the number of reads against other transcripts.

Three of the six aquaporins encoded in the *Ae. aegypti* genome were identified, AAEL003512 (0 reads NBF/2 reads PBM), AAEL005001 (20 NBF/2 PBM) and AAEL005008 (34 NBF/8 PBM). While not significant by our statistical analyses, these numbers may show a trend which supports our previous findings that expression of these aquaporins decreases post-blood meal [61]. We also see a trend which suggests an increase in the presence of oligo- and polypeptide transporter transcripts (Figure 3).

### Immunity-related fat body transcripts

As mentioned previously, the fat body is an important player in mosquito immunity. Synthesis of a variety of anti-microbial peptides to control and erradicate pathogens throughout the mosquito as a whole occurs within this organ [11], [62]. We were able to identify 44 transcripts across our NBF and PBM samples which are thought to encode protein products related to immunity; Table 6 contains selected examples. Included are transcripts thought to encode defensins, cecropins, gram negative and peptidoglycan recognition proteins and dicer, among many others.

**Table 6:** Immune genes expressed in the fat body.

AAEL	NBF	PBM	Vectorbase description
**Expressed in both samples**			
AAEL003849	2	6	Defensin ,Anti-Microbial Peptide Precursor
AAEL009770	30	66	Ubiquitin-conjugating enzyme E2 I
AAEL000621	10	1	Cecropin, Anti-Microbial Peptide.
AAEL003841	10	5	Defensin-A Precursor (AaDef)
AAEL010524	7	5	Hypothetical protein (Putative Tumor Necrosis Factor)
AAEL010466	3	1	Mitogen activated protein kinase kinase kinase 4, mapkkk4, mekk4
AAEL010171	9	3	Peptidoglycan Recognition Protein (Long)
AAEL004675	10	5	Conserved hypothetical protein (Toll pathway related)
AAEL000760	2	6	Clip-Domain Serine Protease, family B.
AAEL006794	1	21	Dicer-1
AAEL006674	30	95	Clip-Domain Serine Protease, family B.
**unique to NBF**			
AAEL007626	3	0	Gram-Negative Binding Protein (GNBP)
**unique to PBM**			
AAEL004142	0	2	Phagocyte signaling-impaired protein

NBF is reads NBF, PBM is reads PBM. Reads were normalized as described in Tables 4 and 5. None were found to have statistically significant differential expression by the R-test we performed. Dicer (AAEL006794) was found to have differential expression (P = 0.026, P<0.05 significant) with DESeq.

**Defensins** are classically known as small (4 kDa) cationic peptides up regulated in response to bacterial challenge in insects. They are capable of forming ion channels into gram positive cells [63]. However, *Ae. aegypti* and *A. gambiae* defensins have been shown to respond to, and have an effect on, infections by certain stages of *Plasmodium*, the causative organism of malaria [63], [64], [65]. We identified two defensins in the fat body with our sequencing (AAEL003849 and AAEL003841) and found them to be present in both our PBM and NBF libraries. They were not found to have statistically significant differential expression.

**Cecropins** are similar to defensins in size and function [66]. In *Ae. aegypti* they have been shown to work in conjuction with defensins against bacterial and *plasmodium* infections [11]. Additionally, cecropins may play a role in *Ae. aegypti's* response to infection with filarial worms, the causative agent of filiariasis, a major health problem in many parts of the world and subject of a World Health Organization erradication program [67], [68]. In our sequencing we identified reads for cecropin (AAEL000621) in our NBF sample and in our PBM sample, but did not find statistically significant differential expression. Increasing sequencing depth in the future would increase both the number of immune related genes identified in the fat body, and provide more data on their differential expression within this organ, pre- and post-bloodmeal.

### q-RT PCR

In order to verify differential expression of selected genes identified via 454 sequencing in the analysis of our libraries, we used quantitative real-time PCR directed at transcripts of interest. Transcripts and results are shown in Table 7 where they are compared with our 454-pyrosequencing data and data from a microarray analysis of blood meal induced transcription changes in whole mosquitoes [36]. Although the qPCR data supports the trends we observed in the analysis of our pyrosequencing results, the levels of change tend to be dissimilar among methods for each gene. We attribute differences in the level of change to the differences in technologies used to obtain gene expression data.

**Table 7:** Comparision of the number of reads in NBF and PBM samples for the transcripts selected for qPCR.

Name	Vectorbase ID	Reads NBF/PBM	Fold change (454 data)	qPCR fold change (ΔΔCt)	Microarray fold change [36]
Vitellogenin	AAEL010434	13/3194	246*	7895	1487
Cathepsin B	AAEL015312	7/3753	536*	529	1347
trypsin	AAEL007818	34/2	0.05*	0.02	0.005
Arginase	AAEL002675	51/1	0.02*	0.02	0.1
Serine-Type Endopepidase	AAEL008781	22/0	-	0.08	0.1
Serine-Pyruvate Aminotransferase	AAEL010480	94/5	0.05*	0.4	0.9

**ΔΔCt:**2∧-((CT sample-CT housekeeping gene1) - (CT calibrator - CT housekeeping gene2)).

CT sample: average CT of 3 qPCR repeats of PBM gene.

CT housekeeping gene1: average CT of 3 qPCR repeats of ribosomal protein S7 PBM.

CT housekeeping gene2: average CT of 3 qPCR repeats of ribosomal protein S7 NBF.

CT calibrator: average CT of 3 qPCR repeats of NBF gene.

*significant, R>9. Trypsin, Arginase and Serine-Pyruvate Aminotransferase also significant by DESeq, p<0.05.

### Conclusions

To date, only a handful of projects describing the transcriptome of *Ae. aegypti* or one of its organs have been reported on. Two projects have utilized microarrays to describe gene expression in whole *Ae. aegypti* mosquitoes [36] or mosquito midguts [69]. To identify early changes post-blood feeding an RNA-seq (illumina) investigation of whole mosquitoes, before and five hours after blood feeding, has also been performed [70]. An earlier transcriptome analysis performed on the *Ae. aegypti* vitellogenic fat body involved a very small number of randomly selected cDNA ESTs from 24 h PBM fat bodies [71]. Our present study now builds upon these investigations by describing in great depth the transcriptome of fat body tissue before blood meal and at the metabolic peak of vitellogenesis after a blood meal has been taken.

In female mosquitoes vitellogenesis involves specific cellular signaling events and transport of large quantities of nutrients in the fat body, an extremely important metabolic and reproductive-associated organ. The transition from a state-of-arrest to vitellogenic fat body is primed by blood feeding and culminates in the deposition of yolk protein into developing eggs. 454 pyrosequencing has enabled us to take a detailed look at the fat body transcriptome of the yellow fever mosquito *Ae. aegypti*, an important disease vector and model organism. The two transcriptomes we compared were considerably different from each other in terms of mRNA expression and reflect the different physiological stages of the fat body in NBF versus PBM mosquitoes undergoing the process of vitellogenesis. This key tissue and its physiological functions are a prime target for novel vector control strategies and our results represent a first in-depth examination of a mosquito fat body transcriptome. In the future, we will extend this study by further analysis of the mosquito fat body transcriptome under different physiological and environmental conditions. One of our long term goals is to identify potential targets for the development of novel insecticides. We suggest that fat body proteins, especially transporters and ion channels, are rational targets for that.

## Methods

### Ethics Statement

The research plan used for this work involving animals was specifically approved by the Institutional Animal Care and Use Committee (IACUC) at New Mexico State University under approval ID #2008-034. All procedures and care are described in the New Mexico State University Animal Care Facility Standard Operating Procedure and on file in the IACUC office there. All persons involved in animal work successfully completed Animal Welfare Training at New Mexico State University and were specifically trained in protocols used in the research plan. All New Mexico State University IACUC care and protocols follow the NIH guidelines described in Guide for the Care and Use of Laboratory Animals: Eighth Edition, ISBN-10: 0-309-15400-6.

### Mosquito Rearing & Blood Feeding

*Aedes aegypti* Rockefeller strain eggs were obtained from MR4 (available as MRA-734) and used to start a laboratory colony [72], [73]. The colony had been maintained in the laboratory for approximately one year at the time the work described in this paper began. Eggs produced by the lab colony were hatched under 25inHg vacuum in 27°C water deoxygenized for 30 minutes. The eggs were left under vacuum for 15 minutes, then eggs and hatched larvae were transferred to pans containing water at 27°C and placed in an incubator maintained at 27°C and 80% relative humidity. Mosquitoes were fed daily a 1∶1∶1 mix of albumin, ground cat food and yeast. Pupae were transferred to a cup containing 27°C water and placed in a cage to hatch. Mosquitoes were maintained with free access to 20% sucrose solution until competent for blood feeding 72 h after emergence.

Mosquitoes were blood fed by placing a live chicken (*Gallus gallus domesticus*) on top of their cage for approximately 30 minutes.

### Total RNA quantities in the fat body tissue PBM

RNA was extracted and purified from fat body tissue (four samples each, NBF and PBM, consisting of 5 fat bodies per sample) and then had concentration measured as described in fat body dissection and RNA isolation.

### Fat body Dissection, RNA Isolation, and poly A+ RNA Purification

200 mosquito abdomens were dissected each from two groups of mosquitoes 72 hours post eclosion and 24 hours post-blood meal in groups of 10. Malpighian tubules, midgut, ovaries and crop were removed, then the abdomens were transferred to eppendorf tubes with 0.5 ml of Trizol reagent (Invitrogen, Carlsbad, CA). They were then homogenized with pellet pestle and handheld homogenizer. Another 0.5 ml of Trizol was added to the cap afterwards and mixed by inversion. RNA was isolated and precipitated following the manufacturer's protocol. RNA pellets were dissolved in RNase-free water. Concentration was measured on a Thermo Scientific nanodrop 1000 (Thermo Scientific). RNA quality was checked using a Bioanalyzer 2100 (Agilent). mRNA isolation was accomplished by separation using poly-A tails with Oligotex solution (Qiagen, Valencia, CA) following the instructions of the manufacturer.

### 454 cDNA library Construction

The cDNA libraries were constructed using the Clontech SMART cDNA Library Construction Kit (Invitrogen) following the manufacturers instructions with modifications [74]. For the reverse transcription, a modified 3′ RT primer: 5′-ATT CTA GAG ACC GAG GCG GCC GAC ATG T_(4)_GT_(9)_ CT_(10)_V N-3′ was used (primer mix: V = A, G or C; N = A, C, G or T). An amplification step was performed using the Advantage 2 PCR kit (Invitrogen) with a modified 3′PCR primer: 5′-ATT CTA GAG GCC GAG GCG GCC GAC ATG T_(4)_GT CT_(4)_G TTC TGT_(3)_ CT_(4)_V N-3′. The first strand was synthesized by combining 3 ul of RNA, (>200 ng), 1 ul modified 3′ RT primer and 1 ul SmartIV oligo (Invitrogen). Tubes were incubated at 72°C for 2 min, cooled to room temperature for 2 min and then combined with 2 ul of 10× first strand buffer, 1 ul of DTT, 1 µl of dNTP mix, 1 ul of MMLV-reverse transcriptase, then incubated at 42°C for 90 minutes. 20 ul of water was added and first strand cDNA stored at −20°C. In a 0.2 ml PCR tube, 2 ul of first strand cDNA, 5 ul of 10× buffer, 1 ul of dNTP-Mix (10 mM), 1 ul of modified 3′ PCR primer (10 um), 1 ul of Advantage Polymerase and 39 ul of water were combined. In an Eppendorf MasterCycler, the following program was run: 94°C−2 min, 24 cycles: 94°C−20 sec , 65°C−20 sec, 68°C−6 min. 50 µg of cDNA was synthesized per experimental sample and purified by phenol/chloroform extraction.

### Sequencing and *De Novo* Assembly

The cDNA library samples described above were run on a 1/× picotitre plate and Roche 454 Genome Sequencer FLX instrument. Binary SFF files generated by the sequencing hardware were converted into fasta, fasta.qual and xml qual files by the sff_extract program [75]. The extracted fasta files and fasta.qual files were then used to perform a *de novo* assembly of the reads using the assembler Mira [28]. The EST and *de novo* options were used to run the assembly software.

### Gene Ontology

Contig files from our assembly were analyzed using Blast2GO. This program uses a pipeline involving a BlastX step followed by gene ontology and annotation steps. For most contigs the default Blast options within Blast2GO were used with the exception of 7 contigs which required lower numbers of results returned due to the size of the results and the maximum size Blast2GO can report. Following Blastx, we performed a sanity check outside of Blast2GO on our contigs by analyzing the species of the best BlastX result for each contig. BlastX results then had gene ontology and annotation steps applied, and were grouped by GO term. Only contigs successfully passing all stages of the pipeline were used in GO term analysis. Level 2 GO terms for each contig were used for our analyses [76].

### Novel Transcript Discovery

To identify potential new transcripts, we aligned our contigs against the reference transcripts available from Vectorbase, using the tool Blat [77]. Those contigs which did not align with the reference transcripts were then aligned with the reference genome using Blat. From this set of contigs (those which did not align with the reference transcripts but aligned with the reference genome) we selected only alignments over 100 bp that matched the genome for at least 75% of the contig. The alignments had to have greater than 99% similarity over the match with the genome and less than 0.7% gaps with the reference genome along the length of the match. Further analysis of these contigs consisted of BlastX at NCBI to identify potential functions from similar protein products if the contigs were to be translated, use of Blast and the genome browser at Vectorbase to verify alignment with the genome and a lack of known protein product, and identification of potential protein domains using SMART [52] and the pfam databases [78].

### Sequence Alignment and Data Analysis

Aligned sequences were generated by using Blat to align generated ESTs to reference transcript sequences from Vectorbase. The aligned output sequences were analyzed for statistically significant differential expression between samples using the R test [37], [38] and a negative binomial distribution was performed with DESeq [79].

### Transporter Identification

From membranetransport.org [80] we retrieved a list composed of 751 putative *Ae. aegypti* membrane transporter genes based on predicted or known amino acid sequences. To this list we added an additional six genes for potential transporters, which we identified through searches of transcript information for *Ae. aegypti* at Vectorbase (AAEL002527, AAEL003136, AAEL006509, AAEL012596, AAEL006650 and AAEL007809). We then searched for and identified matches in our aligned transcript data for the genes on this list.

### Immunity Related Transcript Identification

From our GO results, contigs with the term “immune system process” were selected and their sequences Blasted against the *Ae. aegypti* database at Vectorbase. Results obtained produced e-values typically less than 1e-50, with the majority of results being less than 1e-100, except where noted. Resulting AAEL numbers were then used to look up read numbers and differential expression statistics from our sequence alignment data.

### Quantitative Real-time PCR

Gene-specific primers were developed using Primer BLAST [81]. Total RNA was obtained from fat bodies as described above. Fat body-specific RNAs were isolated after dissection of samples from 30 individual mosquitoes including previtellogenic females 72 h after eclosion and females 24 h post-blood meal. Transcripts were analyzed and quantified with quantitative RT-PCR (qPCR) using iQ Supermix (Biorad, Hercules, CA) and ribosomal protein S7 housekeeping gene as the standard. Primers were as follows: Vitellogenin-A1: Forward primer CTC GTT CCC GCT CTG GCA GC, reverse primer TGT AGC CGC GAC CAA TGT CGG, product length 282; Cathepsin b: Forward primer AGG GTG CAC AGC ACG TAG AGA, Reverse primer TGC CGG AGG TTT CGG GTT GC, Product length 308; Trypsin: Forward primer GCC AAG CTG CAA CGC TGT CC, Reverse primer GGC GCG CAA CAA CGT GTT CA, Product length 449; Arginase: Forward primer GCA ACA TGC TGC GCG GAA AAC A; Reverse primer GCC CAC ATC GCT GCA GTG CT, Product length 449; Serine-Type Endopeptidase: Forward primer AGG TGG CCC TTT TCG AGA CGG A , Reverse primer TGA TTT TCT TCC ACC CGG ATG CAA, Product length 450; Serine-Pyruvate Aminotransferase, Forward primer ACT ACTGAT GGG TCC AGG CCC A, Reverse primer AAG CGA GGC AAC CGT GTC CA, Product length 496; RPS7: Forward Primer TCA GTG TAC AAG AAG CTG ACC GGA, Reverse primer TTC CGC GCG CGC TCA CTT ATT AGA TT. A total of eight RNA samples (4 NBF, 4 PBM) were prepared using the same method for the measurement of total RNA in fat bodies. Samples were then converted into cDNA using an Omniscript RT kit (Qiagen) following the manufacturer's supplied protocol.

## Supporting Information

Figure S1 A and B Size distribution of EST library reads. (A) Library from fat bodies of NBF mosquitoes; (B) Library from fat bodies of mosquitoes 24 h PBM.
(TIF)

Figure S2 A and B. GC content of contigs from NBF (A) and PBM (B) samples.
(TIF)

Figure S3 A and B Blast2GO Blastx results broken down by species NBF(A) and PBM(B).
(TIF)

Table S1 Putative new genes.
(XLS)

Table S2 Percentage of the total number of unique read-transcript alignments (multiple isoforms counted as one transcript).
(XLS)

Table S3 All reads aligned using blat to *Ae. aegypti* reference transcripts from Vectorbase. R-value>9 is considered significant, meaning high likelihood of being differentially expressed between the two conditions.
(XLS)

Table S4 Transcripts reads aligned with using blat, fold change in expression between blood fed and not-blood fed state.
(XLS)
