# Supplementary figures and images for: The Fat Body Transcriptomes of the Yellow Fever Mosquito Aedes aegypti, Pre- and Post- Blood Meal
Source: PLoS One. 2011 Jul 27;6(7):e22573. doi: 10.1371/journal.pone.0022573 (PMC3144915; doi:10.1371/journal.pone.0022573)

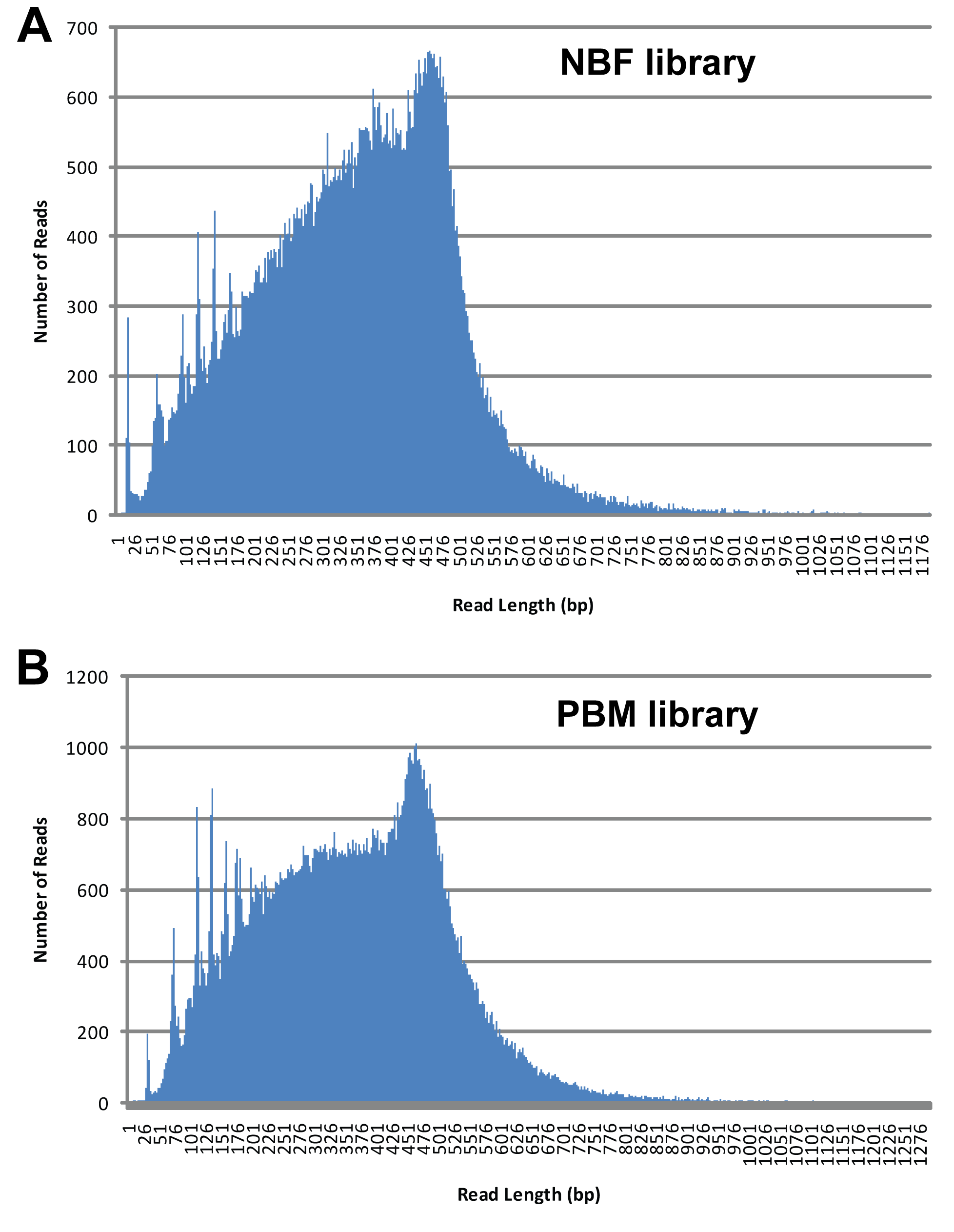

Supplement: Figure S1 — A and B Size distribution of EST library reads. (A) Library from fat bodies of NBF mosquitoes; (B) Library from fat bodies of mosquitoes 24 h PBM. (TIF) [file pone.0022573.s001.tif]

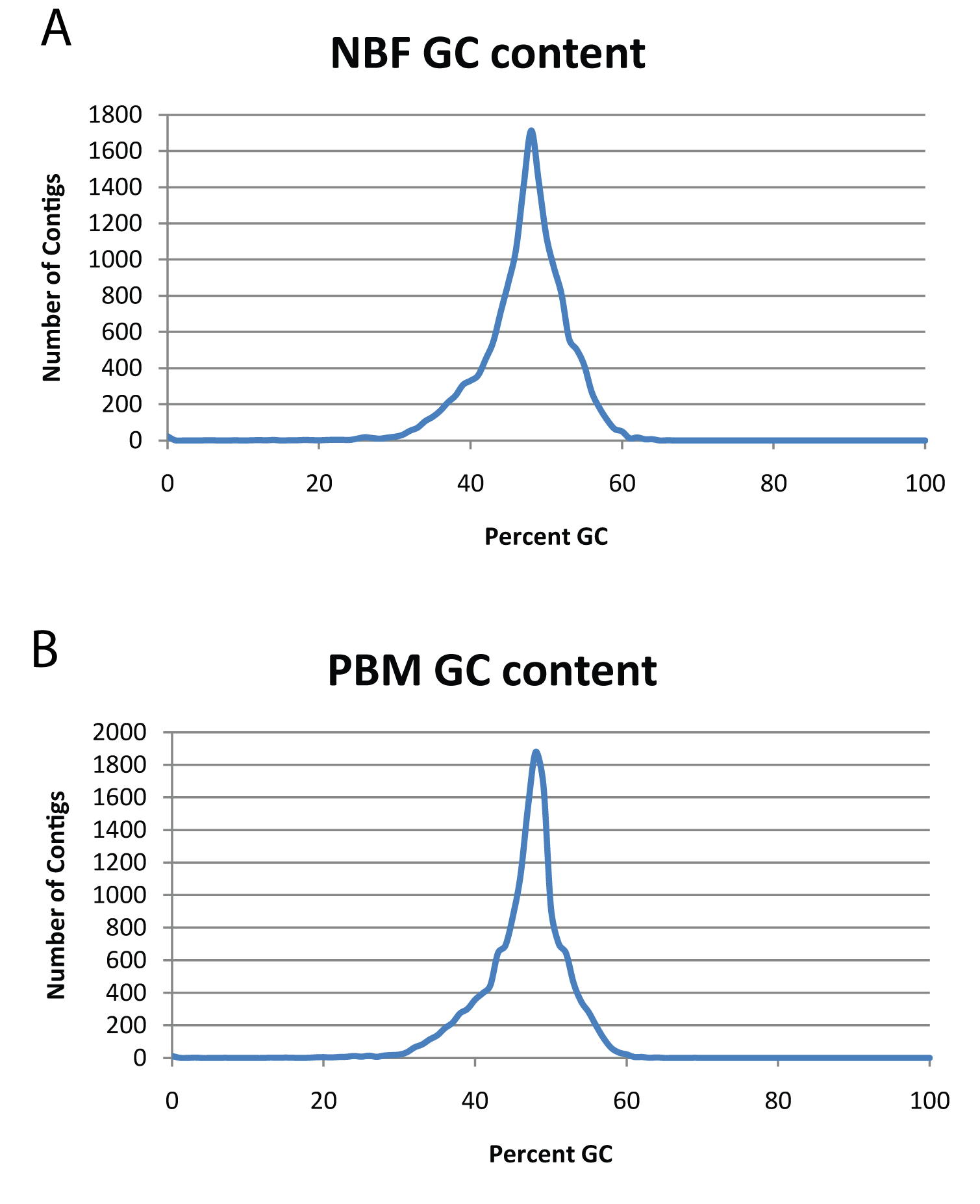

Supplement: Figure S2 — A and B. GC content of contigs from NBF (A) and PBM (B) samples. (TIF) [file pone.0022573.s002.tif]

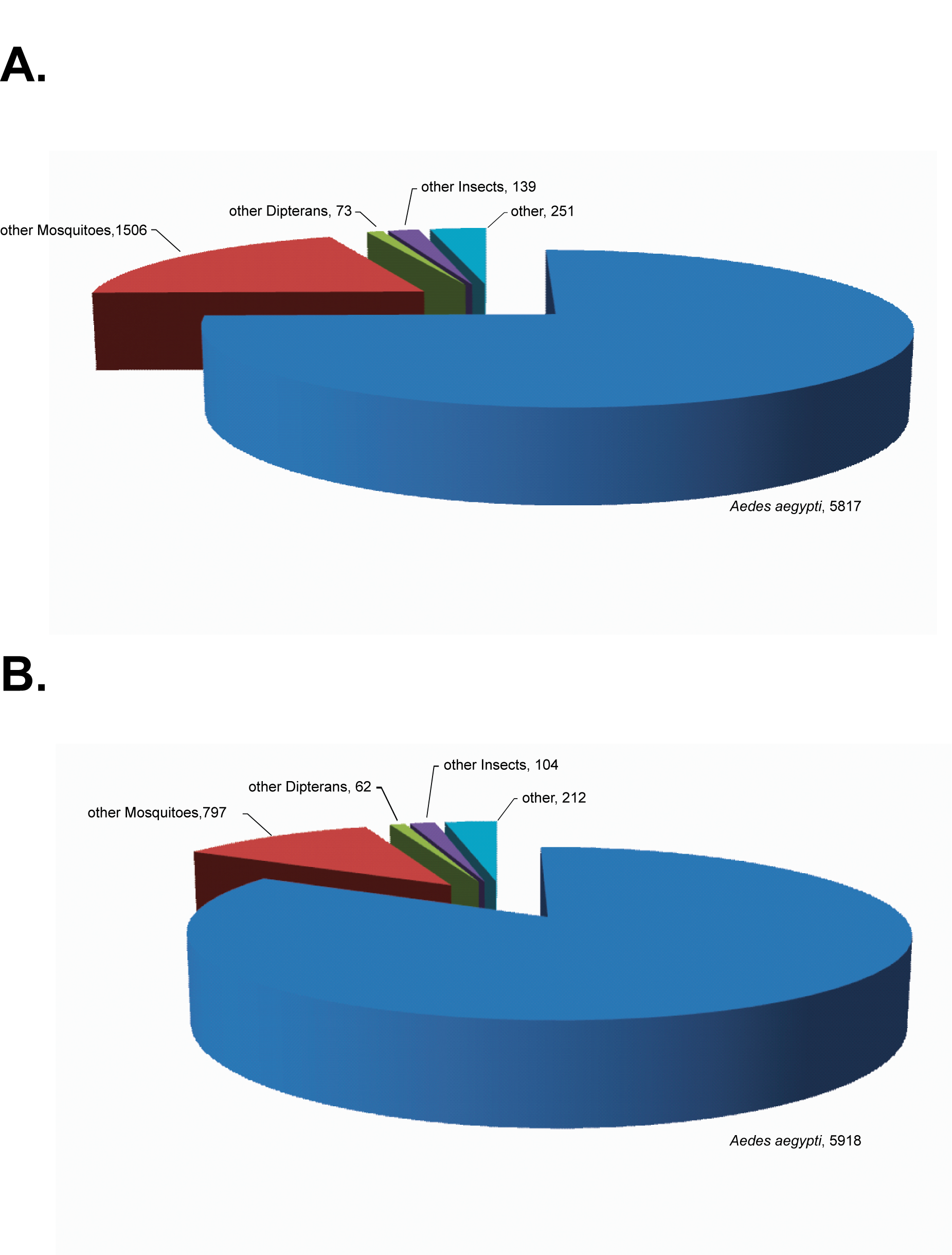

Supplement: Figure S3 — A and B Blast2GO Blastx results broken down by species NBF(A) and PBM(B). (TIF) [file pone.0022573.s003.tif]
